# Pivoting Community-Based Educational Programming for Formal and Informal Caregivers During COVID-19

**DOI:** 10.1093/geroni/igab046.407

**Published:** 2021-12-17

**Authors:** Kara Dassel, Jacqueline Telonidis, Catherine Witt, Linda Edelman

**Affiliations:** 1 University of Utah, Salt Lake City, Utah, United States; 2 University of Utah College of Nursing, Salt lake City, Utah, United States; 3 University of Utah College of Nursing, Salt Lake City, Utah, United States

## Abstract

The Utah Geriatric Education Consortium provides community-based education about Age-Friendly Health Care and Dementia-Friendly Communities targeted towards informal and professional caregivers. As such, we have collaborated with our community partners to host a series of “Fireside Chats”. Since March of 2020, we have hosted 17 Fireside Chats. Our attendance has exceeded our expectations, with over 500 attendees (average of 32 attendees per session). The professional attendees come from a variety of interdisciplinary backgrounds including nursing, medicine, public health, allied health, aging services, and health and long-term care administration. Our non-professional attendees include family caregivers, students, and older adults in the community. This session will address: a) the logistical steps we took (and lessons learned) as we “pivoted” our Fireside Chats into a virtual video-conference format, b) how we redesigned the curriculum to address topics related to COVID-19, and c) will review our evaluation feedback.

